# Infections due to Rare *Cryptococcus* Species. A Literature Review

**DOI:** 10.3390/jof7040279

**Published:** 2021-04-07

**Authors:** Soraya E. Morales-López, Guillermo Garcia-Effron

**Affiliations:** 1 Grupo CINBIOS, Programa de Microbiología, Universidad Popular del Cesar, Valledupar 200002, Colombia; sorayamorales@unicesar.edu.co; 2 Laboratorio de Micología y Diagnóstico Molecular, Consejo Nacional de Investigaciones Científicas y Tecnológicas, Santa Fe 3000, Argentina; 3 Cátedra de Parasitología y Micología, Facultad de Bioquímica y Ciencias Biológicas, Universidad Nacional del Litoral, Santa Fe 3000, Argentina

**Keywords:** *Cryptococcus* sp., cryptococcosis, rare yeast

## Abstract

Infections due to rare *Cryptococcus* species (other than *C. neoformans* species complex, *C. gattii* species complex, *C. albidus* or *C. laurentii)* are barely reported. The aim of this work is to present a comprehensive literature review of all the papers describing infections due to these species referenced in the main databases (PubMed/MEDLINE, ScienceDirect, Scopus, and Google Scholar). Clinical and epidemiological data together with laboratory findings (identification and antifungal susceptibility) of each isolate were analyzed. Fifty-eight cryptococosis due to rare species were described in 46 papers between 1934–2018. These reports included 16 rare *Cryptococcus* spp. that were generally associated with nervous system infections and fungemias. Some species are non-capsulated and are not able to grow at 37 °C. Few species were identified by commercially available methods, making internal transcriber spacer (ITS) and D1/D2 regions sequencing mandatory. The most potent antifungal was amphotericin B (although some species showed high MIC values). The studied strains showed high MICs values to 5-fluorocytosine (all >64 µg/mL), echinocandins (all >8 µg/mL), and fluconazole (>80% of the MICs >4 µg/mL). Due to the scarcity of the data and the absence of guidelines for the treatment of these infections, this review could be informative and could help in the diagnosis and treatment of these infections.

## 1. Introduction

*Cryptococcus neoformans* and *Cryptococcus gattii* are the most common and major pathogenic species complex in the genus *Cryptococcus*. *Cryptococcus (Papiliotrema*) *laurentii* and *Cryptococcus* (*Naganishia*) *albidus* are responsible for near 80% of the reported cases of infection with non-*neoformans*/non-*gatti Cryptococcus* infection [1,2]. The remaining 20% of the infections are caused by other *Cryptococcus* species classically considered saprophytic and non-pathogenic and rarely reported as human pathogens [2].

Those not common species (non *neoformans* species complex, non *gattii* species complex, non *albidus*, non *laurentii) Cryptococcus* are known to be low virulent agents, however, their incidence is rising [1,3,4].

The increased number of these infections may reflect the growing number of patients with risk factors, improvements in the identification of microorganisms, marked medical progress that allow the survival of patients with greater debilitating comorbidities, and the enhanced awareness of such infections by improved laboratory detection of non-typical species [3].

Environmental sources of rare *Cryptococcus* spp. Are highly varied including air, water, living plants, food, and soil. Moreover, some isolates have been recovered from skin or vaginal samples [4,5]. Currently, more *Cryptococcus* spp. are recognized as human pathogens and are considered as emerging yeast pathogens and treatment options for these species are not defined [4,5,6].

The species included in the *Cryptococcus* genus, were conventionally classified based on morphological features, sexual and asexual reproductive structures, and physiological properties. The genus name was originally created to include algae observed from dried scrapings collected from a window sill and then was reassigned it to pathogenic yeasts transferring *Saccharomyces neoformans* into the genus. In 1989, Fell proposed *Cryptococcus neoformans* as the type specie of the genus. In 2015, there were two milestones in the taxonomy of *Cryptococcus*. Firstly, *C. gattii*/*C. neoformans* species complex was divided by Hagen et al. into seven haploid and four hybrid genotypes based on the huge genetic and phenotypic heterogeneity demonstrated in several reports. New names were proposed replacing the known serotype and genetic (RFLP genotypes) varieties. *C. neoformans* and *C. deneoformans* replace *C. neoformans* var. *grubii* (Serotype A, VNI, VNII, and VNIII) and *C. neoformans* var. *neoformans* (Serotype D, VNIV), respectively. On the other hand, *C. gattii* (serotypes B and C) was divided into 5 species: *C. gattii* (VGI), *C. deuterogatii* (VGII), *C. bacillisporus* (VGIII), *C. tetragattii* (VGIV), and *C. decagatii* (VGIV/VGIIIc) [7]. Secondly, an extensive taxonomy revision of the genus *Cryptococcus* was performed by Liu et al. and several emendations were done after a complete molecular phylogenetic analysis [8]. It was noted that species inclusion criteria in the genus *Cryptococcus* was highly artificial. The genus contains species with multiple evolutionary histories and distributed in all five major lineages within the *Tremellomycetes* (*Cystofilobasidiales*, *Filobasidiales*, *Tremellales*, *Trichosporonales*, and *Holtermannia*), where they are sometimes amalgamated with species of other anamorphic or teleomorphic genera [8]. After these revisions, the main human pathogens of the old genus *Cryptococcus* were reclassified as the named 7 haploid species of the old *C. neoformans*/*C. gattii* complex (and the four hybrid), *Papiliotrema laurentii* (*C. laurentii*), and *Naganishia albidus* (*C. albidus*) [8]

The aim of this work is to present a comprehensive literature review of clinical cases of infections due to *Cryptococcus* species other than *C. neoformans* species complex, *C. gattii* species complex, *N. albidus* (*C. albidus*), or *P. laurentii* (*C. laurentii*). The 2015 taxonomy revision will be used through this work [8].

## 2. Materials and Methods

Literature search strategy and selection criteria: A comprehensive search of the literature on PubMed/MEDLINE, ScienceDirect, Scopus, and Google Scholar of clinical cases due to each of the 70-accepted species of *Cryptococcus* genus described in the book Yeast [9] and that correspond to the species validated until 2005 was performed. Later, a second search for reports of clinical cases including the newest *Cryptococcus* species validated from January 2006 to June 2020 was done. For this last search, the keyword “*Cryptococcus* sp. nov” was added and the newest genus emends were also revised [8]. Abstracts from different conferences from 2000 onwards were also accessed. Of each case, data on gender and age of the patient, clinical specimen where the strain was isolated, clinical manifestations, treatment, clinical outcome, taxonomical identification method used for identification of the isolate, and the antifungal susceptibility profile of each strain were included in the analysis. 

Subsequently, publications about the taxonomy and the phylogeny that had been selected as theoretical support of this research were reviewed. Finally, references in each manuscript were revised to identify additional cases of non-*C. neoformans*/*C. gattii/C. albidus/C. laurentii* yeast infections. All papers were accessed and read.

## 3. Results

### 3.1. Rare Cryptococcus spp. As Agents of Human Mycoses

As for November 2020, fifty-eight clinical cases reporting rare *Cryptococcus* spp. infections were included in this analysis. These cases were described in 46 individual papers. These reports included 16 rare *Cryptococcus* spp., distributed as follows: *Cryptococcus* (*Naganishia*) *adeliensis* (n = 3 cases), *Cryptococcus* (*Cutaneotrichosporon*) *arboriformis* (3), *Cryptococcus* (*Filobasidium*) *chernovii* (1), *Cryptococcus* (*Cutaneotrichosporon*) *cyanovorans* (3), *Cryptococcus* (*Cutaneotrichosporon*) *curvatus* (6), *Cryptococcus* (*Naganishia*) *diffluens* (4), *Cryptococcus* (*Papiliotrema*) *flavescens* (1), *Cryptococcus* (*Naganishia*) *friedmannii* (1), *Cryptococcus* (*Vanrija*) *humicola/humicolus* (16), *Cryptococcus* (*Naganishia*) *liquefaciens* (3), *Cryptococcus* (*Hannaella*) *luteolus* (3), *Cryptococcus (Cystofilobasidium*) *macerans* (1), *Cryptococcus* (*Filobasidium*) *magnus* (3), *Cryptococcus* (*Solicoccozyma*) *terreus* (1), *Cryptococcus* (*Filobasidium*) *uniguttulatum* (8), and *Cryptococcus* (*Naganishia*) *uzbekistanensis* (1). From now on, the new genus names will be used but the terms “rare Cryptococcus spp.” will be used to refer to all these species to make it more understandable. The oldest data correspond to cases of *F. uniguttulatum* (1934), *H. luteolus* (1956), and *F. magnus* (1960) infections [10,11,12], while the most recent data correspond to reports of *F. magnus* (2018), *C. curvatus* (2018), and *C. cyanovorans* (2018) [3,13,14]. 

### 3.2. Infectious Diseases and Epidemiology

Rare *Cryptococcus* spp. were isolated in patients with different immune status. Among the described patients, 81% (n = 47) had a concomitant condition associated with immunosuppression including cancer [4], continuous ambulatory peritoneal dialysis [15,16], diabetes [15,17,18], lymphoma [16,19,20], myeloma [19], leukemia [21], cystic fibrosis [14], rheumatoid arthritis [1], Sezary syndrome [22], AIDS [23,24], and major surgeries or solid tumors [19,25]. 

Rare *Cryptococcus* spp. were generally associated with nervous system infections (meningitis, meningoencephalitis, myeloradiculitis, ventriculitis) and fungemias. They were also associated, but to a lesser extent, with peritonitis, pneumonia, vaginitis, eye infections (keratitis, conjunctivitis, ophthalmopathy), nail infections (melanonychia and onychomycosis), urinary tract infection, and tenosynovitis. Overall, 44.8% (n = 26) of the strains, representing 13 out of the 16 species included in this review, were isolated from normally sterile sites (all but *F. chernovii*, *F. magnus*, and *N. friedmannii*). These two *Filobasidium* spp. are phylogenetically close while the described *Naganishia* sp. is the species of this genus with the furthest common ancestor of the *Naganishia* genus included in this review. These rare *Cryptococcus* spp. were mostly isolated from cerebral spinal fluid (CSF) [4,21,22,23,24,26,27,28,29,30], blood and skin lesions [2,25,31,32,33,34,35], peritoneal fluid [15,36], but also from vagina [2,3,35,37], nails [16,35,38,39], bone marrow [20], urine [19,40], nasal cavities [41], or oral cavity [42]. 

The age range of the patients described in the publications included in this work was from 4 to 83 years (average 46) and 70% were men. *V. humicola*, *N. diffluens*, *H. luteolus*, *N. adeliensis*, *F. chernovii*, and *C. curvatus* were the only species found in children and teenagers (7 years-old, 17 years-old, and 4 years-old, and three pediatric (no data), respectively). 

**Figure 1:**
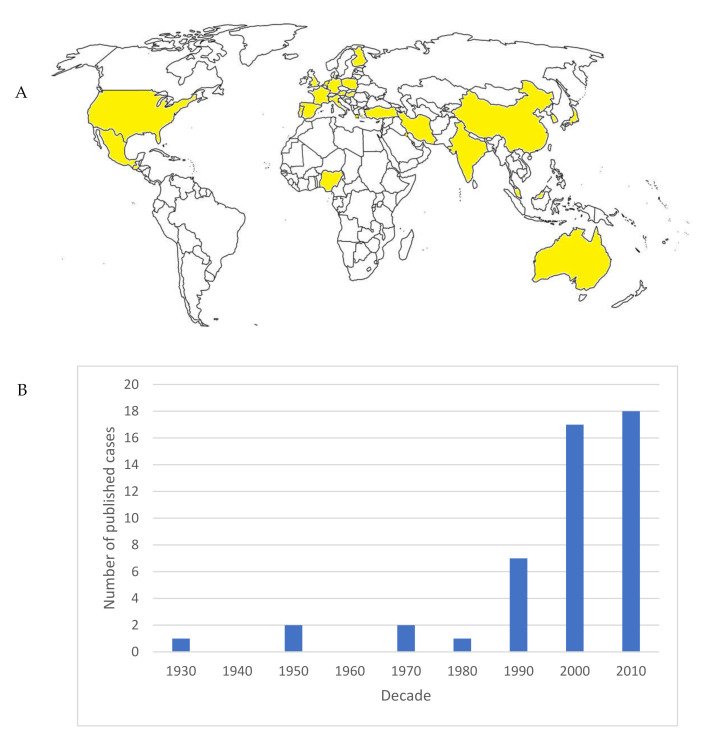
(**A**) Map showing the countries were rare *Cryptococcus* spp. isolates were reported (highlighted in yellow). (**B**) Published cases per decade.

The cases were mostly reported in European (Germany, Hungary, The Netherlands, Spain, France, Finland, Portugal, Austria, Poland, United Kingdom, Italy, Greece) and Asian (Iran, Turkey, China, Japan, Korea, Kuwait, Malaysia, India) countries. There were few cases in Oceania (Australia) and North and Central America (United States, Mexico, Guatemala), while there was only one report from Africa (Nigeria). There were no records in South America (Figure 1).

### 3.3. Laboratory Diagnosis and Taxonomical Identification

#### 3.3.1. Direct Examination, Indian Ink, and Serology

Some of the rare *Cryptococcus* spp. included in this revision were described as non-encapsulated yeasts in clinical samples; as an example, we can highlight *N. adeliensis*. Using India ink smears to analyze CSF obtained from patients with *N. adeliensis* infections, few nonencapsulated rounded yeast cells were observed, latex agglutination test (glucuronoxylomannan capsule antigen) were negative and non-encapsulated blastospores were observed on rice agar [21]. Other species, as *F. magnus*, were described as spherical to ellipsoidal capsulated yeasts [41] or as round to oval cells which can present true hyphae or pseudohyphae without capsule in Indian ink smears [3].

On the other hand, *F. chernovii* isolates were described as capsulated, oval to elongated yeasts forming chains of budding cells [41]. *N. diffluens* is described as globose to elliptical, capsulated cells of different size [43]. Other species, as *N. liquefaciens*, showed ovoid cells with narrow capsules and negative latex agglutination test results [28,44].

#### 3.3.2. Taxonomical Identification. Are Classical and Commercially Available Phenotypical-Based Methods Able to Identify the Rare Cryptococcus Species?

After analyzing the published data, it is clear that molecular identification is mandatory to reach an unequivocal taxonomical classification of these rare *Cryptococcus* species. Sequencing of the internal transcribed spacer (ITS1 and ITS2) together with D1/D2 regions of the rDNA were the methods of choice for molecular identification [1,13,15,20,22,28] (Table 1). Despite this fact, only 43.4% of the strains (25 out 58) included in this review were identified by molecular methods. Most of the strains (21 out of 33) identified by phenotypic-based methods were reported earlier than 2002. It has to be highlighted that all the reported *V. humicula* were not identified by molecular-based methods [2,19,24,25,26,34,35,38,45,46]. Oppositely, all the *C. arboriformis* and *Naganishia* spp. were identified by sequencing [1,15,20,21,28,33,37,40,42,43,44].

**Figure 2:**
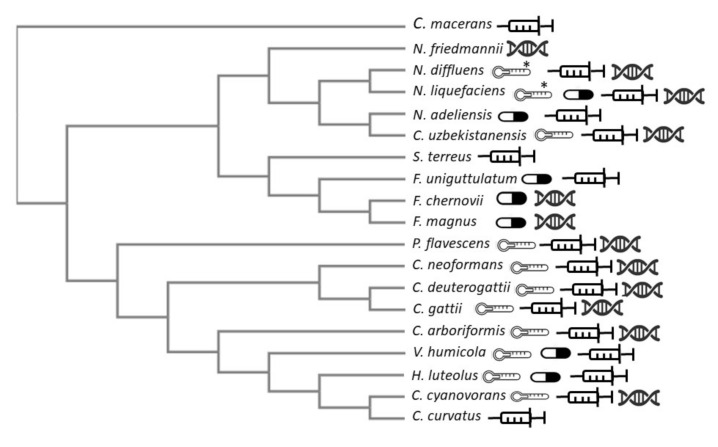
Neighbor-joining tree without distance corrections obtained using the clustal omega tool (https://www.ebi.ac.uk/). The following Genebank LSU D1/D2 sequences were used: FJ534907 (*Cryptococcus deuterogattii* CBS 10514T), AF075526 (*Cryptococcus gattii* CBS 6289T), FJ534909.1 (*Cryptococcus neoformans* CBS 8710), AB260936 (*Cutaneotrichosporon arboriformis* CBS 10441T), AF189834 (*Cutaneotrichosporon curvatus* CBS 570T), JF680899 (*Cutaneotrichosporon cyanovorans* CBS 11948T), NG_059011.1 (*Cystofilobasidium macerans* CBS 10757T), AF181530 (*Filobasidium chernovii* CBS 8679T), AF181851 (*Filobasidium magnum* CBS 140T), AF075468 (*Filobasidium uniguttulatum* CBS 1730T), AF075482 (*Hannaella luteola/luteolus* CBS 943T), AF137603.1 (*Naganishia adeliensis* CBS 8351T), AF075502 (*Naganishia diffluens* CBS 160T), AF075478 (*Naganishia friedmannii* CBS 7160T), AF181515 (*Naganishia liquefaciens* CBS 968T), AF181508 (*Naganishia uzbekistanensis* CBS 8683T), AB035042 (*Papiliotrema flavescens* CBS 942T), AF075479 (*Solicoccozyma terreus* CBS 1895T), AF189836 (*Vanrija humicola* CBS 571T). The thermometer graphic represents the species able to grow at 37 °C. * represent poor growth at 37 °C. Pill graphics show species with the highest fluconazole MIC (>16 ug/mL). Syringe graphs show species isolated from normally sterile sites. Double strand graphs show the species identified by molecular-based methods.

Only *C. cyanovorans*, *P. flavescens*, *V. humicola*, *H. luteolus*, and *N. uzbekitanensis* were able to grow at 37 °C while all the other mentioned rare *Cryptococcus* species grow at 28–30 °C [11,14,15,19,20,43] (Figure 2). This characteristic may explain why the identification of these rare *Cryptococcus* species is difficult since most of the phenotypically-based tests are usually performed at 37 °C. Looking at the phylogenetic tree (Figure 2), the species related to *C. neoformans*/*C. gattii* grow better at 37 °C than the other rare *Cryptococcus* species.

The rare *Cryptococcus* species isolated from human specimens belong mostly to the *C. albidus* clade (*Naganishia adeliensis*, *Naganishia diffluens*, *Naganishia liquefaciens*, *Filobasidium magnus*, and *Naganishia uzbekitanensis*) [20,21,28,45] and were formerly misidentified as *Cryptococcus albidus*. Similarly, *Papiliotrema flavescens*, which belong to the *C. laurentii* complex, were initially misclassified as *C. laurentii* [27].

Some species such as *H. luteolus* or *F. unigutulattus* were easily identified by commercially available systems (ID 32 and Vitek 2) where these species are classified as *Cryptococcus luteolus* and *Cryptococcus unigutulattus*, respectively [11,18]. In the cases of *F. chernovii*, *N. liquefaciens*, and *F. magnus*, the identification differed depending on the used method: *F. chernovii*, was identified as *Cryptococcus albidus* if API-ID 32 C^®^ and as *Cryptococcus unigutulatus* if API 20 C or Vitek was used; *N. liquefaciens* was identified as *Rhodotorula minuta* and *C. albidus* or not identifiable by ID 32C and *F. magnus* was identified as *Cryptococcus laurenti* (API 20 C) or *Cryptococcus unigutulattus* (Vitek) [41].

Thus, phenotypic-based methods as API 20 C, ID32C, VITEK2, etc., could identify only 5 out of the 16 described rare species as *Cryptococcus* spp. including: *C. curvatus*, *V. humicula*, *H. luteolus*, *C. macerans*, and *F. uniguttulatus* [2,17,18,22,23,25,26,29,33,45] (Figure 2). For this reason, the use of molecular methods is mandatory (ITS and/or D1/D2 regions sequencing) [1,14,15,20,33,42,47,49].

### 3.4. Antifungal Susceptibility Testing

The revised literature includes reports from 1934 to 2018. Thus, different methods of antifungal susceptibility testing were used: microdilution, disk diffusion, e-test ^®^, Sensititre^®^, VITEK^®^, etc. We decided to include in our analysis the reports published later than 2000 since standardized methods were available (although some results were obtained using older versions of the CLSI and/or EUCAST documents). Considering these facts, we will be analyzing the in vitro activities of echinocandins, amphotericin B (AMB), azoles, and 5-fluorocytosine (5FC) against rare *Cryptococcus* species. It is long known that the members of the echinocandin class are inactive against Basidiomycetes [50]. However, eight reports [1,2,22,23,25,26,40,42] show data regarding susceptibility testing of echinocandins against several *Cryptococcus* species, as expected, all the tested strains showed very high MIC values to all echinocandins. Turning to 5FC, most of the species showed high MIC values (≥64 µg/mL) except for some *C. arboriformis*, *N. diffluens*, *C. curvatus*, and *V. humicola* strains (all with ≥8 µg/mL). On the other hand, a wide range of AMB MIC values (0.06 to 8.00 µg/mL) were observed but only 4 isolates showed AMB MIC values ≥2.00 µg/mL (*C. arboriformis*, *N. diffluens*, *V. humicola*, and *N. liquefaciens*, one each).

Azole agents MICs varied depending more on the studied drug than in the species and/or genus analyzed. No phylogenetic relationship was observed between antifungal susceptibility and genus/species (Figure 2). For FLC, the MIC (µg/mL) range was wide, showing values between 0.06 and >256 µg/mL. For PSC between 0.015 and >8, for ITC between 0.03 and >32, and for VRC was 0.03- and >8. Overall, fluconazole (FLC) showed the poorest activity (very high MICs values, 80% of the strains showed MIC ≥4.00 µg/mL) [2,4,5,21,22,26,28,34,35,40,48,49] (Figure 2) followed by itraconazole (ITC) and voriconazole (VRC). The highest FLC MIC values were observed for *Filobasidium* spp., *Naganishia* spp., and *V. humicola* (Figure 2). Posaconazole (PSC) was correctly tested only for eight strains (three *V. humicola*, two *C. curvatus*, and one each of *N. liquefaciens* and *F. unigutulattum*) [2,28,40,48] and showed good activity against all but the *N. liquefaciens* strain which showed very high MIC values for all tested azole drugs (>256, >16, >8, and >8 µg/mL for FLC, ITC, VRC, and PSC, respectively) [28]. For ITC, four isolates showed high MICs including the formerly described *N. liquefaciens* [28], one *F. magnus* [41], one *F. unigutulattum* [48], and one *F. chernovii* [41] (ITC MICs: 1.00 µg/mL for the last three species). The azole drug that showed the lowest MIC values was VRC. All the species showed low CIM values (less than 0.5 µg/mL) [1,2,13,15,21].

Except for one strain of *N. diffluens* and one strain of *V. humicola,* data about the susceptibility profile of this atypical species show that echinocandins and 5FC were inactive. AMB was the most active agent in vitro (MIC range 0.03 and 8 µg/mL) (Table 2).

### 3.5. Antimicrobial Therapy

As for August 2020, there are no interpretive minimum inhibitory concentration clinical breakpoints for *Cryptococcus* spp. except for *C. neoformans* and AMB by using the EUCAST microdilution method. On the other hand, there are epidemiological cut off values and several antifungal agents for *C. neoformans* (VND), *C. gattii* (VGI), and *C. deuterogatti* (formerly C. *gattii* VGII) [51,52]. Despite scarce published data, some studies suggest that these rare species may be less susceptible (in vivo) to antifungals than *C. neoformans* species complex [21,35].

Overall, AMB was the most frequently used antifungal agent administered as a single drug or in combination with 5FC. However, this statement may be biased if one takes into account that many of the papers included in this study were published before the azole era. The duration of therapy varied widely (from one to 11 weeks) [1,14,17,21,43] or with maintenance therapies of up to one year [26,29].

Because the data are scarce and in the absence of guidelines for the treatment of these species infections, this review could be informative and could help to understand the epidemiology of these infections.

### 3.6. Brief Description of the Clinical Reports due to Rare Cryptococcus spp. Divided by Species (In Alphabetic Order of Genus and Species)

#### 3.6.1. *Cutaneotrichosporon* spp.

##### *Cutaneotrichosporon arboriformis* 

Sugita et al. (2007) presented a *Cryptococcus* (*Cutaneotrichosporon*) *arboriformis* strain isolated from urine samples of a 73-year-old man diagnosed with chronic renal failure. There was no other information about the strain susceptibility, clinical data, or evolution [40]. Seven years later, Im et al. described a case of peritonitis in a 58-year-old man with diabetes mellitus, hypertension, and continuous ambulatory peritoneal dialysis for 10 months. This strain was susceptible to AMB and FLUC (Vitek 2 AST-YS01 system) and the treatment included intravenous AMB (0.5 mg/kg/day) for 4 weeks and oral fluconazole (400 mg/day) for 3 weeks [15].

In 2015, Hadano et al. published the case of a 60-year-old woman with central line-associated bloodstream infection, rheumatoid arthritis, and diabetes mellitus with septic shock complicating septic bursitis of the left elbow. The strain was recovered from retroculture. The patient was treated with liposomal AMB despite the strain showed elevated AMB MIC values when tested in vitro. The other tested antifungals were micafungin, VRC, and ITC. As expected for any basidiomycete, this strain was not susceptible to the tested echinocandin. On the other hand, the isolate showed low MIC values for both tested azole drugs [1].

All the described strains were identified by sequencing of the ITS and D1/D2 regions since phenotype-based methods (ID32C and Vitek 2 YST systems) were not able to differentiate this species.

##### *Cutaneotrichosporon curvatus* 

Dromer et al. in 1995 described the first infection due to *C. curvatus* in a 30-year-old patient, diagnosed with AIDS and mieloradyculitis. *Cryptococcus curvatus* was isolated from the CSF and it was identified by ID 32 C systems. There is no antifungal susceptibility data. The patient received FLC 400 mg/day and later, AMB (0.7 mg/kg/day) for seven days and maintenance for two months, but he died after refusing to continue treatment [23].

In 2006, a fatal peritonitis was described in a Polish 39-year-old male patient with gastric lymphoma that was unsuccessfully treated with AMB [16]. On the other hand, in 2010, Bernal-Martinez described the susceptibility of 2 *C. curvatus* recovered from vaginal samples of two patients. The isolates were identified by fermentation of carbon sources and assimilation tests, as well as the evaluation of other morphological and physiological characteristics. They showed high micafungin, anidulafungin, and caspofungin MIC values and low ITC, VRC, PSC, and AMB MIC values. There is no additional information on these isolates or on whether the vaginal exudates were considered or not infections [2].

Recently, Jeng et al. described a polymicrobial keratitis case in an immunocompetent 54-year-old woman. In that case, the strain was recovered from corneal scrapes and identified by sequencing of the D1-D2 and ITS regions. Furthermore, the patient received several antifungal therapy schemes that included AMB, FLC, VRC, and 5FC, focal cryotherapy of the abscess, and chlorhexidine 0.02% drops [13].

##### *Cutaneotrichosporon cyanovorans* 

In 2013, Knox et al. described the first clinical report of *C. cyanovorans* infection. The strain was recovered from lung nodules in a 61-year-old diabetic man with rheumatoid arthritis and a diagnosis of pneumonia. However, this description was made in an abstract the ASM (Micro) 2013; but it was not published later and there are no data of sensitivity or outcome of this patient [47].

Recently, Van der Bruggen et al. described two women (37 and 20 years-old) with cystic fibrosis in which *C. cyanovorans* were recovered from respiratory samples (throat swabs, sputum, and bronchoalveolar lavage). Both patients showed pancreatic insufficiency and liver diseases related to their cystic fibrosis disease. None of the strains were identified by the MALDI-TOF system (Bruker) and the definitive identification was made by ITS and ribosomal large subunit sequencing. Antifungal susceptibility testing was performed by using the Yeast One system. The strains showed high echinocandin and 5FC MIC values (>8 and 64 µg/mL, respectively) and slightly high FLC MICs (8 µg/mL). Oppositely, these isolates showed low AMB, VRC, ITC, and PSC MIC values. The 37-year-old woman was treated for six months with VRC after bilateral lung transplantation; while the 20-years-old patient did not receive any antifungal treatment [14].

#### 3.6.2. *Cystofilobasidium* spp.

##### *Cystofilobasidium macerans* 

In 1997, Lindsberg et al. described the only reported case of a human infection due to *C. macerans* in a 24-year-old male patient diagnosed with meningoencephalitis. In this case, the fungus was isolated from CSF and identified by carbohydrate assimilation and fermentation tests. The strain grew at a temperature of 28–30 °C, but not at 35–37 °C. There were no data of antifungal susceptibility of the strain. The patient received FLC therapy (200 mg a day) for a week followed by an oral regimen of 150 mg FLC/day [29].

#### 3.6.3. *Filobasidium* spp.

##### *Filobasidium chernovi* 

Currently, the only report corresponds to the fungal colonization of the nasal cavity of a pediatric patient with cancer in Kuwait. In this case, three identification systems were used: ID32C, API 20 C AUX and Vitek ^®^ that yielded as identification *Cryptococcus albidus*, *Cryptococcus unigitulattus*, and *Cryptococcus unigutulattus*, respectively. Finally, DNA sequencing of internally transcribed spacer ITS-1 and ITS-2 and D1/D2 regions of allowed the definitive identification of the isolate as *Cryptococcus* (*Filobasidium*) *chernovi*. The strain grew at 28–30 °C, but not at 35–37 °C. It showed high caspofungin, anidulafungin, 5FC, ITC, and FLC MIC values and low AMB, PSC, and VRC MIC values. Susceptibility testing was performed by using E-test [41].

##### *Filobasidium magnus* 

In 1960, Castellani described the colonization of the cutaneous ulcer of a leg in an 81-year-old male patient, and in that moment, the strain was identified as *Cryptococcus ater* and conserved as CBS 4685 [12]. Later, this strain was reclassified as *Cryptococcus* (*Filobasidium*) *magnus* by means of its ribosomal DNA sequence.

In 2011, Khan et al. described two *F. magnus* isolates, isolated from the nasal cavity of two cancer patients. When using ID32C, API 20 C, and Vitek identification systems, both strains were identified as *Cryptococcus laurentii* and *Cryptococcus unigutulattu*, respectively. The sequencing of ITS regions allowed the final and correct identification of *F. magnus.* Both isolates grew at 28 °C, but did not at 37 °C and exhibited high MIC values (32 µg/mL) for anidulafungin, caspofungin, and 5FC, and low MIC values for PSC (0.5 µg/mL), VRC (0.12 µg/mL), AMB (0.75 µg/mL), and FLC (8 µg/mL). These susceptibility data were obtained by the E-test method [41].

Recently, Ghajari et al. described a *F. magnus* strain isolated from a vaginal secretion of a 23-year-old inumonocompetent patient with vulvovaginitis. Identification was done by sequencing of ITS regions and the isolate showed low FLC, ITC, and 5FC MIC values [3].

##### *Filobasidium uniguttulatus* (*unigutulata*)

The first clinical report of an infection due to *C. uniguttulatus* was in 1934. In that year, Zach et al. described a *Eutorulosis uniguttulata* isolated from the nails of a 48-year-old male patient with onychomycosis [10].Then, in 1977, Kwon-Chung et al. described two *C. uniguttulatus* strains in the fingernail and throat of two patients. In that case, both strains grew at 30 °C, but not at 37 °C [39].

In 1989, Gugnani et al. studied the incidence of yeasts in pregnant and non-pregnant women in Nigeria and a strain of *C. uniguttulatus* was recovered from the vagina and cervix of a non-pregnant asymptomatic woman [36].

McCurdy described a ventriculitis in a 65-year-old woman with ovarian cancer caused by a *C. uniguttalatus* recovered from CSF and identified by the ID 20C system. It shows high FLC and 5FC MIC values (64 µg/mL for both antifungals), and low MIC values for AMB and ITC (0.25 and 1.0 µg/mL, respectively). The patient was treated with AMB (0.6 mg/kg) and 5FC; but three months later, she died by a subarachnoid hemorrhage [4].

García Martos et al. described *C. unigutulattus* in the vagina and nails from two Spanish patients, both isolates were identified by the ID 32 system and showed elevated MIC values for FLC and 5FC (64 µg/mL) and low AMB MIC values (0.25 µg/mL). These data were obtained by using the CLSI M27 A document and Sensititre Yeast One ^®^ [35]. Another strain was described by Manzano-Gayosso et al. (2008), who studied the onychomycosis agent incidence in type 2 diabetes mellitus patients [18].

In 2011, Pan et al. described the first case of *C. uniguttulatus* meningitis in a 37-year-old male patient. In this case, the strain exhibited high FLC and 5FC MIC values (64 µg/mL) and low ITC, PSC, VRC, and AMB MIC values (1.0, 0.5, 0.5, and 0.125 µg/mL, respectively) obtained by CLSI M27 A3. The patient received AMB as treatment [48].

Another *C. uniguttulatus* meningitis case was described by Animalu et al. in 2015 in a 72-year-old woman with Sezary syndrome, lung carcinoma (with previous resection), and adrenal insufficiency. *C. uniguttulatus* was recovered from CSF, and identified with the API 20 C system. As the strains isolated by Pan et al., the isolate showed high FLC and 5FC MIC values to fluconazole (>64 µg/mL) and low MIC values for ITC, PSC, VRC, and AMB (0.5, 1.0, 0.25, and <0.12 µg/mL, respectively) [22].

#### 3.6.4. *Hanaella* spp.

##### *Hanaella luteolus* (*luteola*) 

In 1956, Binder et al. described an infection in a 4-year-old female patient diagnosed with primary pulmonary cryptococcosis (Toruloma). The isolated yeast was identified using biochemical tests as *C. luteolus* [11]. Later, García Martos et al. (2002) reported the susceptibility profile of a *C. luteolus* strain isolated from a respiratory secretion of a female patient. The strain exhibited high 5FC MIC values (64 µg/mL), and low FLC, ITC, and AMB MIC values (4.0−8.0, 0.06−0.03, and 0.12–0.25 µg/mL obtained by CLSI M27 A/Sensititre methods, respectively) [35].

The last clinical *C. luteolus* report is the one by Hunter et al., in 2014, in a 68-year-old man with type II diabetes mellitus and tenosynovitis. In this case, the yeast was identified as *C. luteolus* (Vitek and DNA sequencing) and the treatment consisted of one year of oral FLC 800 mg/day [17].

#### 3.6.5. *Naganishia* spp.

##### *Naganishia adeliensis* 

In 2004, Rimek et al. reported a fatal case of meningitis in a 40-year-old woman with a diagnosis of acute myeloid leukemia and allogeneic peripheral blood stem cell transplantation. The yeast was recovered from CSF; it grew at 30 °C; but not at 37 °C and it was identified as *Cryptococcus albidus* by the ID 32 C system. The sequences of the internal transcribed spacer ITS 1 and ITS 2 and the D1/D2 region of the 26S rDNA were obtained and the strain was identified as *Cryptococcus adeliensis*. MIC was determined by CLSI microdilution (M27-A) and e-test methods and by both, the strain showed a high MIC to FLC, 5FC and low AMB and VRC MICs. The patient received 5 mg of liposomal AMB/kg/day; after 2 days, 120 mg of 5FC/kg was added, and after 3 days, additional AMB at a dose of 0.25 mg was intrathecally administered every three days [21].

In 2005, Tintelnot et al. reexamined nine isolates previously identified as *Cryptococcus albidus* by ID 32 C. Using ITS 1 and ITS 2 and the D1/D2 regions sequencing as identification tools, two of those strains were identified as *Cryptococous adeliensis*. Both strains were isolated from the oral cavity of a HIV+ female and from a lung biopsy of a male progressive cancer patient. No information regarding treatment and outcome of these patients were obtained. Both isolates could grow at 30 °C, but not at 35 °C [42].

##### *Naganishia diffluens* 

Several strains of *C. diffluens* were isolated from the skin of patients with atopic dermatitis and in healthy individuals. They were wrongly identified as *Cryptococcus albidus* by ID 32 C systems. Later, DNA sequence analysis of internal transcribed spacer regions and the D1/D2 26S rRNA gene, allowed the reclassified the clinical isolates as *C. diffluens* [31,49].

Kantarcioğlu et al. (2007) described the first case of subcutaneous cryptococcosis due to *C. diffluens* in a 17-year-old man with skin lesions. The isolate was not identified by API32C and ITS regions and the D1/D2 domains sequencing was required. The strain grows better at 30 °C than at 37 °C. It showed high AMB and 5FC MIC values (8 and 16 µg/mL, respectively) and low FLC and ITC MIC values (1 and 0.25 µg/mL). The patient received 100 mg/day ITC therapy and complete resolution was reported after three months [43].

In 2011, Zhan et al., characterized the fungal skin microbiota in patients with atopic dermatitis and in healthy subjects and described *C. diffluens* on the skin of both populations. In those cases, the identification was made by D1/D2 large subunit sequencing [33].

##### *Naganishia friedmannii* 

The only reported clinical case due to *C. friedmannii* is a 57-year-old immunocompetent man with an onychomycosis after a nail trauma. The strain was identified by DNA sequencing as *C. friedmanii* and the susceptibility testing was performed by using the CLSI M27 document. The obtained MIC values were 0.25 µg/mL for FLC and AMB and 0.125 µg/mL for ITC. The patient was treated satisfactorily with oral ITC (200 mg daily) [37].

##### *Naganishia liquefaciens* 

In 2003, Sugita et al. described the skin colonization by *C. liquefaciens* in patients with atopic dermatitis and in healthy patients [31]. Subsequently, Conde-Pereira et al. (2015) described a fatal polymicrobial meningitis in a 31-year-old woman due to a yeast firstly identified as *C. albidus* (ID 32 C and Malditof MS systems) that was reclassified as *C. liquefaciens* by ITS region sequencing. The strain showed high MIC values to all antifungals tested by using Sensititre Yeast One ^®^ method [28].

In this same year, Takemura et al. described a central venous catheter-related fungemia in a 71-year-old man caused by a yeast initially identified as *Rhodotorula minuta* and *C. albidus* by ID 32 C system. Later, and using ITS sequencing, the identification was corrected as *C. liquefaciens*. The isolate showed high 5FC MIC values (>64 µg/mL) and AMB (2.0 µg/mL) and low MIC values for FLC (4.0 µg/mL), ITC (0.25 µg/mL), and VRC (0.125 µg/mL). Antifungal susceptibility testing was performed by following the CLSI M27 A3 document. The patient received VRC treatment that was later switched to FLC [44].

##### *Naganishia uzbezistaniensis* 

There is only one *C. uzbezistaniensis* clinical report published so far. The strain was isolated from a bone marrow aspirate in an 83-year-old man with lymphoma. In this case, the strain was not identified by the Vitek system and the sequencing of the D1/D2 region was the only method that allowed the identification. No susceptibility data were reported. The patient received high-dose of FLC. The patient died due to an acute necrotizing tracheobronchial pulmonary aspergillosis [20].

Recently, Ghajari et al. described a *C. magnus* strain isolated from a vaginal secretion of a 23-year-old immunocompetent patient with vulvovaginitis. Identification was done by sequencing of ITS regions and the isolate showed low FLC, ITC, and 5FC MIC values [3].

#### 3.6.6. *Papiliotrema* spp.

##### *Papiliotrema flavescens* 

In 1998, Kordosis et al. described a 34-year-old woman with AIDS and meningitis due to *C. laurentii* (CBS 8645 strain). The strain was identified by ID 32 C. Antifungal susceptibility testing was performed by agar diffusion (E-test method) and the obtained results were 0.25 µg/mL for AMB, 0.5 ug/mL for ITC, 1.25 (5 fluorocytosine) and 4 (FLUC). Later, the strain was re-identified as *C. flavescens* based on rDNA sequence analyses. Later, it was noticed that the D1/D2 sequence of this strain differs by five nucleotide substitutions from that of other *C. diffluens* (e.g., CBS 942). This issue may require further studies to establish the final taxonomic status of the described strain [27].

#### 3.6.7. *Solicoccozyma* spp.

##### *Solicoccozyma terreus* 

The only *C. terreus* isolate was described by Méndez-Tovar et al. from Mexico in a male patient with AIDS and meningitis in 1995. There were neither susceptibility data nor report on the outcome of the patient [30].

#### 3.6.8. *Vanrija* spp.

##### *Vanrija humicolus* (*humicola*)

The first human infections owed to *C. humicolus* were described in patients with conjunctivitis and ophthalmopathy, in 1975 [46]. Later, Velez et al. described a 55-year-old male patient with melanonychia of both big toes who received systemic ITC (200 mg twice a day) for 7 days during the first week of each month, for 3 months (ITC pulse treatment). In this case, strain grew well at 30 °C and were identified with the API 20 C system; however, there are no susceptibility data [38].

In 1997, Rogowska et al. described the first case of meningitis due to *C. humicolus* in a 31-year-old male with AIDS with drug abuse history. There was no information neither on the identification method used nor on the susceptibility profile of the isolate. The patient was treated with FLC (400 mg intravenously plus oral 4 weeks of oral treatment). He later died [24].

Ryder et al. [34], and Gasca et al. [19], both in 1998, reported the recovery of *C. humicolus.* Ryder et al. described a cutaneous isolate identified by the API 20 C system with a FLC MIC value of 4 µg/mL., while that Gasca et al. described four strains from different clinical samples (sputum, urine, stomach secretion, bronchial exudate). In this last report, two patients had a solid tumor, one had myeloma, and the other had a lymphoma. The isolates were identified as *Candida humicola*, by the API 20 C system and there were no susceptibility data.

García-Martos et al. described strains isolated from skin samples with low AMB and ITC MIC values (0.5–1.0 µg/mL and 0.12–0.25 obtained with CLSI M27 and Sensititre methodologies, respectively) [35].

Shinde et al. described a systemic cryptococcosis due to *C. humicolus*. The strain was isolated from blood, bone marrow, liver biopsy, lymph node, and urine, in a 7-year-old boy from India. In this case, all the strains were identified by the ID 32 C system and molecular identification was not performed. The patient was successfully treated with intravenous liposomal AMB combined with FLC [45].

Baka et al. (2007) described a *C. humicolus* fungemia in an HIV-negative immunocompromised patient who had undergone sigmoidectomy and colostomy. The identification was carried out by the system ID 32C. Antifungal susceptibility testing was performed by CLSI and Etest methods. The strain showed low MIC values to azoles, 5FC, and AMB (MIC values were not reported). The patient received intravenous AMB, VRC, and FLC treatments for three weeks [25].

In 2010, Bernal-Martínez et al. [2], described 3 skin and 1 blood *C. humicolus* isolates. All the strains showed high echinocandin and 5FC MIC values (micafungin, anidulafungin, and caspofungin MIC values >16 µg/mL and 8.0–64.0 µg/mL for 5FC). On the other hand, MIC values for FLC, AMB, VRC, PSC, and ITC showed wide ranges (2.0–16.0 µg/mL, 0.03–1.0 µg/mL, 0.01–0.25 µg/mL, 0.03–0.50 µg/mL, and 0.06–1.0 µg/mL, respectively). This data were obtained by microdilution with Eucast E Def 7.1. In our knowledge, the last clinical report of *C. humicolus* infection is a fatal meningitis of a 49-year-old man diagnosed with pulmonary cryptococcosis and meningitis described by Ramli in 2012. The strain showed high MIC values to all tested antifungals (FLC, ITC, caspofungin, and AMB). The patient received intravenous AMB (0.7 mg/kg/day) as induction therapy for 6 weeks, followed by FLC 400 mg/12 h for eight weeks [26].

## 4. Discussion

We consider as rare *Cryptococcus* spp. those species not included into the *C. neoformans* and *C. gattii* complexes and not identified as *Naganishia albidus* or *Papiliotrema laurentii*. We found 58 clinical cases were the most common reported species were *V. humicola/humicolus* (n = 16), *F. uniguttulatum* (n = 8), *C. curvatus* (n = 6), and *N. diffluens* (n = 4), followed by *N. adeliensis*, *C. argoriformis*, and *C. cianovorans* with 3 reports each.

These species have been mostly isolated in Europe and Asia from human infections since 1934. As it happens for the most common *Cryptococcus* spp., these rare species were isolated from CSF, blood, and skin. These infections are more common in middle age (media 46 years-old) male patients.

Some important issues have to be highlighted in terms of diagnostics of infections due to these species. The most important are that some of these species were described as not-encapsulated yeasts in clinical samples. Subsequently, these species showed negative agglutination tests (negative capsule antigen) and no studies were performed using the newest lateral flow devices able to detect *Cryptococcus* capsular antigens. This false negative capsular antigen detection was also described for some *C. gattii* isolates [53,54,55]. Another important point to consider is the inability of most of the rare *Cryptococcus* spp. to growth at 35–37 °C. This fact can explain why these species are barely isolated since most of the clinical laboratories incubate their CSF samples at this temperature. This inability to growth at physiological temperature raised doubts about the real pathogenic capacity of some of the species. However, 9 of the 16 species described in this review were isolated from a normally sterile site, confirming that they were the etiological agents of proven invasive fungal diseases, although some of them do not grow in vitro at 37 °C.

In addition, a wrong identification using phenotypic-based methods could contributed to the low prevalence of these species since molecular identification is needed to be able to correctly identify them. This fact would be the main limitation of this review, thus the taxonomical identification in around 30% of the described strains relies only in phenotypical methods. As an example, all the *V. humicolus* and all but one *F. uniguttulatus* included strains were identified only by carbon-assimilation-based methods [4,22,24,26,32,38,39,45,48]

As other *Basidiomycetes* and specifically the most common *Cryptococcus* spp. echinocandins are inactive against them. Most of the reported strains have high 5FC MIC values. The MIC values for the polyene AMB were strain dependent, meaning that wide AMB MIC values ranges were encountered and the values varied within the same species. FLC was the azole with the poorest in vitro potency (80% of the strains showed MIC ≥ 4.00 µg/mL) while VRC showed the lowest MIC values (≤0.5 µg/mL for all species). However, the data were obtained using different methods, some of them obsolete. As a consequence of these analysis, we can conclude that antifungal susceptibility testing should be performed in the event of an infection by these pathogens.

Turning to the most common therapy used in the clinical setting to treat these infections, AMB stands out. It was used alone or in combination with 5FC despite that this last antifungal has little or no in vitro activity. However, it has to be taken into account that many of the reported cases were published in the last century (pre-azole era).

After the analysis of the literature, several knowledge gaps were identified. The areas that need to be addressed to be able to combat rare *Cryptococcus* spp. infections are:

- To establish the real burden of these fungal diseases.
- To confirm whether or not the capsular antigen is negative just for the absence of capsule in certain *Cryptococcus* spp. or because the used antibodies are extremely *C*. *neoformans* specific.
- To establish which of the molecular identification procedures is better to identify correctly these species (which gene/DNA region or genes/DNA regions are more informative).
- To perform a study of the antifungal susceptibility of these species using contemporary standardized methodologies in order to recognize intrinsic resistant species within these rare *Cryptococcus* spp.

Despite the recognition of the knowledge gap, due to the scarcity of the data and the absence of guidelines for the treatment of these infections, this review could be informative and could help in the diagnosis and treatment of these infections.

## Figures and Tables

**Table 1:** Rare *Cryptococcus* spp. infections: Epidemiological data, isolation samples, and identification methods.

Rare *Cryptococcus* Species	Gender	Age (Years)	Clinical Sample	Taxonomical Identification Methods	ITSD1/D2 Sequencing	Growth at:		Reference
						37°	28–30 °C	
***Cutaneotrichosporon arboriformis***	F	60	Blood	ID 32 C	Done	NA	NA	[1]
	M	58	Peritoneal fluid	Vitek	Done	pos	NA	[15]
	M	73	Urine	ND	Done	NA	NA	[40]
***Cutaneotrichosporon curvatus***	F	NA	Vagina	Biochemical tests	ND	NA	NA	[2]
	M	30	CSF	ID 32 C	ND	NA	pos	[23]
	M	39	peritoneal fluid	NA	NA	NA	NA	[16]
	F	54	Corneal scrapes	ND	Done	neg	pos	[13]
***Cutaneotrichosporon cyanovorans***	M	61	Respiratory sample	ND	Done	NA	NA	[47]
	F	37	Respiratory sample	MaldiTof	Done	pos	NA	[14]
	F	20	Respiratory sample	MaldiTof	Done	pos	NA	
***Cystofilobasidium macerans***	M	24	CSF	Biochemical tests	ND	neg	pos	[29]
***Filobasidium chernovii***	NA	NA	Nasal cavities	ID 32 C/API 20 C/Vitek	Done	neg	pos	[41]
***Filobasidium magnus***	M	81	Skin	ND	Done *	NA	NA	[12]
	F	23	Vagina	ND	Done	NA	pos	[3]
	NA	NA	Nasal cavities	ID 32 C/API 20 C/Vitek	Done	neg	pos	[41]
***Filobasidium unigutulattum***	NA	NA	Nail	API 20 C/Vitek	Done	NA	NA	[18]
	M	37	CSF and endobronchial biopsy	ND	Done	neg	pos	[48]
	F	65	CSF	ID 20 C	ND	NA	NA	[4]
	F	NA	Vagina/cervix	Biochemical tests	ND	NA	pos	[36]
	F	NA	Vagina/nails	ID 32 C	ND	NA	NA	[35]
	F	72	CSF	ID 20 C	ND	NA	NA	[22]
***Hanaella luteolus***	F	4	Bronchial swabs	Biochemical tests	ND	pos	pos	[11]
	F	NA	Respiratory sample	ID 32 C	ND	NA	NA	[35]
	M	68	Surgical samples	Vitek	Done	NA	NA	[17]
***Naganishia adeliensis***	F	40	CSF	ID 32 C	Done	neg	pos	[21]
	F	NA	Throat swabs	ID 32 C	Done	neg	pos	[42]
	M	NA	Lung biopsy	ID 32 C	Done	neg	pos	
***Naganishia diffluens***	NA	NA	Skin	ND	Done	NA	32	[31]
	NA	NA	Skin	ND	Done	NA	NA	[33]
	M	17	Skin	API 20 C	Done	poor	pos	[43]
***Naganishia friedmannii***	M	57	Nail	ND	Done	NA	pos	[37]
***Naganishia liquefaciens***	F	31	CSF	ID 32 C/MALDI-TOF	Done	NA	NA	[28]
	NA	NA	Skin	ND	Done	NA	32	[31]
	M	71	Blood	ID 32 C	Done	NA	NA	[44]
***Naganishia uzbekitanensis***	M	83	Bone marrow aspirate	Vitek	Done	pos	NA	[20]
***Papiliotrema flavescens***	F	34	CSF	ID 32 C	Done	pos	pos	[27]
***Solicoccozyma terreus***	M	NA	CSF	API 20 C/ID 32 C or Vitek	ND	NA	NA	[30]
***Vanrija humicola***	NA	NA	Stomach secretion	API 20 C	ND	pos	NA	[19]
	NA	NA	Urine	API 20 C	ND	pos	NA	
	NA	NA	Sputum	API 20 C	ND	pos	NA	
	NA	NA	Bronchial swabs	API 20 C	ND	pos	NA	
	NA	NA	Skin	API 20 C	ND	NA	pos	[34]
	M	31	CSF	ND	ND	NA	NA	[24]
	NA	NA	Skin	ID 32 C	ND	NA	NA	[35]
	NA	NA	Conjunctiva	NA		NA	NA	[46]
	NA	NA	Skin (3)/Blood (1)	ID 32 C	ND	NA	NA	[2]
	M	7	Blood, Bone marrow, liver biopsy, lymph node, urine	ID 32 C	ND	NA	NA	[45]
	M	55	Nail	API 20 C	ND	NA	pos	[38]
	M	39	Blood	ID 32 C	ND	NA	NA	[25]
	M	49	CSF	ID 32 C	ND	NA	NA	[26]

NA: Not available. ND: Not done. F: Female. M: Male. ITS: Internal transcribed spacer. Includes ITS1-5.8S-ITS2 regions of the rDNA. * In the original paper by Castellani et al. [12], no ITS sequencing was performed. However, the strain was later sequenced and conserved in the CBS-Fungal Biodiversity Center as *Cryptococcus magnus* (later reclassified as *F. magnus*).

**Table 2:** Antifungal susceptibility, treatment, and outcome of the studied *Cryptococcus* spp.

*Rare Cryptococcus* Species	MIC (µg/mL) ^a^									AST ^b^ Method	Treatment	Outcome	Year of Publication	Country of Isolation	Ref.
	FLC	ITC	PSC	VRC	MCF	ANF	CSF	5FC	AMB						
*Cutaneotrichosporon arboriformis*	ND	0.06	ND	0.125	>16	ND	ND	4	2	CLSI M27A3	L-AMB	Survived	2015	Japan	[1]
	<1	ND	ND	ND	ND	ND	ND	8	0.5	Vitek 2 AST-YS01	AMB 0.5 mg/kg/day for 4 weeks and oral FLC 400 mg/day for 3 weeks	Survived	2014	Korea	[15]
	ND	ND	ND	ND	ND	ND	ND	ND	ND	ND	NA	NA	2007	Japan	[40]
*Cutaneotrichosporon curvatus*	4.0–8.0	0.25–1.0	0.06	0.06–0.25	>16	>16	>16	1.0–64.0	0.25	Eucast E Def 7.1	NA	NA	2010	Spain	[2]
	ND	ND	ND	ND	ND	ND	>16	ND	ND	ND	FLC 400 mg/day AMB, 0.7 mg/kg/day for seven days and maintenance for two months.	Died (2 months later)	1995	France	[23]
	ND	ND	ND	ND	ND	ND	ND	ND	ND	ND	AMB	Died	2006	Poland	[16]
	ND	ND	ND	ND	ND	ND	ND	ND	ND	ND	NA	NA	2003	Japan	[49]
	ND	ND	ND	0.25	ND	ND	ND	ND	0.5	CLSI M27 A3	Topical AMB 0.15% drops 2 hourly along with oral FLC 400 mg once daily, topical VRC 1% drops 4/day	Survived	2018	United Kingdom	[13]
*Cutaneotrichosporon cyanovorans*	ND	ND	ND	ND	ND	ND	ND	ND	ND	ND	NA	NA	2013	Australia	[47]
	8	0.25	0.5	0.25	8	8	8	>64	1	Sensititre Yeast One	VRC	Survived	2018	The Netherlands	[14]
	8	0.5	1	0.5	8	8	8	>64	1	Sensititre Yeast One	No treatment	Survived			
*Cystofilobasidium macerans*	ND	ND	ND	ND	ND	ND	ND	ND	ND	ND	FLC (200 mg/day) 1 year.	Survived	1997	Finland	[29]
*Filobasidium chernovii*	>256	>32	1	0.75	ND	>32	>32	>32	0.023	E-test	NA	NA	2011	Kuwait	[41]
*Filobasidium magnus*	ND	ND	ND	ND	ND	ND	ND	ND	ND	ND	NA	NA	1960	Portugal	[12]
	0.062	0.031	ND	ND	ND	ND	ND	ND	0.062	CLSI M27 S3	Oral KTC 200 mg oral/day/10 days	Survived	2018	Iran	[3]
	8.00–24.00	1.00−4.00	0.5–1	0.12–0.19	ND	>32	>32	>32	0.75	E-test	NA	NA	2011	Kuwait	[41]
*Filobasidium unigutulattum*	ND	ND	ND	ND	ND	ND	ND	ND	ND	ND	NA	Survived	2008	Mexico	[18]
	>64	1	0.5	0.5	ND	ND	ND	>64	0.125	CLSI M27 A3	Intravenous AMB (0.7 mg/kg/1 day) plus oral 5FC (100 mg/kg/day) for 11 weeks	Survived	2011	China	[48]
	64	1	ND	ND	ND	ND	ND	>64	0.25	CLSI M27 T	0.6 mg/kg AMB and 5FC	Survived	2001	USA	[4]
	ND	ND	ND	ND	ND	ND	ND	ND	ND	ND	NA	NA	1989	Nigeria	[36]
	64–256	0.5–2	ND	ND	ND	ND	ND	>64	0.25–1	CLSI M27 A Sensititre Yeast One	NA	NA	2002	Spain	[35]
	128	0.5	1	0.25	ND	R ^c^	R ^c^	>64	0.125	ND	VRC4 months	Survived	2015	USA	[22]
*Hannaella luteolus*	ND	ND	ND	ND	ND	ND	ND	ND	ND	ND	NA	Survived	1956	Hungary	[11]
	4.0–16.0	0.03–0.25	ND	ND	ND	ND	ND	>64	0.125–0.50	CLSI M27A/Sensititre Yeast One	NA	NA	2002	Spain	[35]
	ND	ND	ND	ND	ND	ND	ND	ND	ND	ND	800 mg FLC for one year	Survived	2014	USA	[17]
*Naganishia adeliensis*	32/>256	0.25/2.00	ND	0.25/0.125	ND	ND	ND	>32/>64	0.125/0.094	CLSI M27 A/E-test	L-AMB 5 mg/kg/day, 120/kg 5FC and intrathecall AMB 0.25 mg/72 h	Died	2004	Alemania	[21]
	ND	ND	ND	ND	ND	ND	ND	ND	ND	ND	NA	NA	2005	Alemania	[42]
*Naganishia diffluens*	ND	ND	ND	ND	ND	ND	ND	ND	ND	ND	NA	NA	2003	Japan	[31]
	ND	ND	ND	ND	ND	ND	ND	ND	ND	ND	NA	NA	2011	Japan	[33]
	<1	0.25	ND	ND	0.5	ND	ND	16	8	CLSI M27 A	ITC 100 mg/day	Survived	2007	Turkey	[43]
*Naganishia friedmannii*	0.25	0.125	ND	ND	ND	ND	ND	ND	0.25	CLSI M27 A3/S3	Oral ITC 200 mg/day	Survived	2017	Iran	[37]
*Naganishia liquefaciens*	>256	>16	>8	>8	ND	ND	ND	>64	1	Sensititre Yeast One	AMB (0.7 mg/kg/day)	Died	2015	Guatemala	[28]
	ND	ND	ND	ND	ND	ND	ND	ND	ND	ND		NA	2003	Japan	[31]
	4	0.25	ND	0.125	ND	ND	ND	>64	2	CLSI M27 A3	VRC and profilaxis with FLC 100 mg/day	Survived	2015	Japan	[44]
*Naganishia uzbekitanensis*	ND	ND	ND	ND	ND	ND	ND	ND	ND	ND	High-dose FLC	Died	2011	USA	[20]
*Papiliotrema flavescens*	4	0.50	ND	ND	ND	ND	ND	1.25	0.25	E-test	AMB (40 mg day) and 5FC (10 g day)	Survived	1998	Greece	[27]
*Solicoccozyma terreus*	ND	ND	ND	ND	ND	ND	ND	ND	ND	ND	NA	NA	2015	Mexico	[30]
*Vanrija humicola*	ND	ND	ND	ND	ND	ND	ND	ND	ND	ND	NA	NA	1998	Mexico	[19]
	ND	ND	ND	ND	ND	ND	ND	ND	ND	ND	NA	NA			
	ND	ND	ND	ND	ND	ND	ND	ND	ND	ND	NA	NA			
	ND	ND	ND	ND	ND	ND	ND	ND	ND	ND	NA	NA			
	4	ND	ND	ND	ND	ND	ND	ND	ND	CLSI M27 A	NA	NA	1998	Several Countries	[34]
	ND	ND	ND	ND	ND	ND	ND	ND	ND	ND	Intravenous FLC 400 mg plus oral FLC for 4 weeks	Died	1997	Poland	[24]
	ago-16	0.12–0.5	ND	ND	ND	ND	ND	2.0–16	0.5–1	CLSI M27 A		NA	2002	Spain	[35]
										Sensititre Yeast One					
	ND	ND	ND	ND	ND	ND	ND	ND	ND	ND	NA	NA	1971	Italy	[46]
	2.0–16.0	0.06–1.0	0.03–0.50	0.01–0.25	>16	>16	>16	8.0–64.0	0.03–1.0	Eucast Def 7.1	NA	NA	2010	Spain	[2]
	ND	ND	ND	ND	ND	ND	ND	ND	ND	ND	L-AMB plus FLC	Survived	2004	India	[45]
	ND	ND	ND	ND	ND	ND	ND	ND	ND	ND	Sistemic ITC (200 mg twice a day/7 days/3 month	Survived	1996	Spain	[38]
	S ^c^	S ^c^	S ^c^	S ^c^	ND	ND	R ^c^	S ^c^	S ^c^	CLSI M 27	AMB+VRC+FLC for 3 weeks	Survived	2007	Greece	[25]
	R ^c^	R ^c^	ND	ND	ND	ND	R ^c^	ND	R ^c^	E-test	AMB (0.7 mg/kg/day); FLC 400 mg twice per day for 8 weeks	Died	2012	Malasia	[26]

^a^ MIC values separated by hyphens indicate ranges. MIC values separated by bars depict results obtained by different AST methods and are in the same order that the method described in the AST method column. ^b^ AST: Antifungal susceptibility testing. CLSI: Clinical Laboratory Standards Institute. EUCAST: European Committee on Antimicrobial Susceptibility Testing. M27T: CLSI M27 tentative document, CLSI M27 A: approved document. M27S3: CLSI M27 supplemental document 3. NA: Not available ND: Not done. MIC: Minimal inhibitory concentration. FLC: fluconazole. ITC: itraconazole. PSC: posaconazole. VRC: voriconazole. MCF: micafungin. ANF: anidulafungin. CSF: caspofungin. 5FC: 5-fluorocytosine. AMB: amphotericin B. L-AMB: liposomal amphotericin B. KTC: ketoconazole. ^c^ There are no clinical breakpoints for Cryptococcus spp. thus these results are wrongly informed in the original paper. However, we included the data considering the scant.

## Data Availability

No new data were created or analyzed in this study. Data sharing is not applicable to this article.
